# Lived experiences and coping strategies of persons seeking infertility treatment in the Kumasi metropolis: a descriptive phenomenological study

**DOI:** 10.1186/s12905-023-02194-6

**Published:** 2023-02-20

**Authors:** Louisa Annan-Frey, Edward Appiah Boateng, Alberta Lomotey, Christopher Lartey, Veronica Dzomeku

**Affiliations:** 1 Nursing and Midwifery Training College, Dunkwa-On-Offin, Ghana; 2 Department of Nursing, Kwame Nkrumah University of Science and Technology, Kumasi, Ghana

**Keywords:** Infertile couple, Gender, Lived experience, Infertility treatment, Coping strategies

## Abstract

**Background:**

Women seeking fertility treatment face myriad challenges that they must adapt and adjust to daily. This aimed at exploring the experiences and coping strategies of such persons in the Kumasi.

Metropolis.

**Methods:**

A qualitative approach was employed and a purposive sampling technique was used to select 19 participants. A semi-structured interview was used to collect data. The data collected were analyzed using Colaizzi's method of data analysis.

**Results:**

Persons living with infertility had emotional experiences of anxiety, stress, and depression. Socially, participants experienced isolation, stigma, societal pressure, and marital problems due to their inability to conceive. The key coping strategies adopted were spiritual (faith-based) and social support. Though formal child adoption can be an option, no participant preferred it as a coping strategy. Some participants also reported using herbal medicine before going to the fertility centre upon realizing that the approach was not helping in achieving their desired outcome.

**Conclusion:**

Infertility is a source of suffering for most women diagnosed with it, resulting in significant negative experiences in their matrimonial homes, families, friends, and the community at large. Most participants rely on spiritual and social support as their immediate and basic coping strategies. Future research could evaluate the treatment and coping strategies and also determine the outcomes of other forms of treatment for infertility.

**Supplementary Information:**

The online version contains supplementary material available at 10.1186/s12905-023-02194-6.

## Background

The condition infertility is the inability of couples to procreate after one year or more of regular unprotected sexual intercourse and is estimated to affect over 72 million couples worldwide [1, 2]. In Africa, the prevalence of infertility is as high as 30% and varies from region to region, even within the same country [3]. Despite the fact that both males and females suffer from infertility, in many African societies, particularly Ghana, the populace often pressures females more than males for their childlessness. Also, males in such situations are often discrete about their situation. This mostly presents a difficult and painful grieving process for women living with infertility in such settings [4]. Women with infertility endure the conflux of individual, interpersonal, social, and devout issues, bringing a sense of disappointment to them [5]. It is a potential cause of marital instability in numerous African societies where childbearing is used as a parameter for a successful marriage. This is often because marriage without children is full of pressure from family members and society and may eventually lead to divorce due to the frustrations the couples encounter [6, 7].

Among married people with fertility problems, their best treatment option is assistive reproductive technology and innovation (Craftsmanship). These methods incorporate in-vitro fertilization (IVF), Gamete intra-fallopian transfer (GIFT), embryo cryopreservation [8], surgery for men with infertility and cryopreservation of oocytes for patients with cancer as well as adoption and surrogate motherhood [8]. Seeking fertility counselling boosts the management of childlessness to make married people succeed. Women who go for fertility counselling cope well with childlessness and have more psychological support than those who do not [10].

Societal pressure on women living with infertility can be enormous in our part of the world, making them feel that they need divine intervention [11]. This causes significant emotional and psychological stress for affected women. For instance, women who are infertile in the Ekiti Region of South West Nigeria are often given the outcast treatment and are buried outside the town along with those who suffered mental illness when they die [12].

In Ghana, the predominance of primary and secondary barrenness is 2% and 14% respectively. The predominance of infertility is 11.8% among women and 15.8% among men [14]. Married women with no live birth are more likely to be those seeking infertility treatment as voluntary childlessness is not common in the country [15]. In fact, childbearing is presumed to be the main determinant of the union between a man and a woman [16]. Thus, marriage and childbirth move together. Tabong and Adongo [17] explain that modernization has not weakened the deep-rooted tradition of having a child as soon as possible after marriage. Hence, all couples prefer to have children. This preference is crucial in understanding and treating couples with infertility. At the time of data collection, the costs for In vitro fertilization (IVF) and Intrauterine Insemination (IUI) treatments in Ghana were about forty-five thousand Ghana cedis (USD 5000) and thirty-two thousand Ghana cedis (USD 3556) respectively.

Couples seeking fertility treatment are always worried about their condition [13]. Despite the high predominance of the condition in Ghana, and the proof that infertility is a major reproductive issue with a huge social problem, works on infertility conducted within the Kumasi Metropolis have not focused on the experiences among women seeking treatment for infertility. Rather, the priority of these studies has been on fertility regulation than infertility [18, 19]. Population control, as a priority, has often negated the impact of involuntary childlessness [20]. Moreover, sociocultural factors shape the lives and experiences of those living with infertility [21]. This study, therefore, sought to explore the lived experiences and coping strategies of women seeking treatment for infertility in three selected hospitals in the Kumasi Metropolis, Ghana.

## Methods

### Study design

Heidegger's hermeneutical phenomenology research approach was used in this study. The design enabled researchers to have a deeper understanding of the lived experiences and coping strategies of women living with infertility in the Kumasi Metropolis.

### Study setting

This work was conducted in three hospitals that were purposively selected because they run fertility clinics in the Kumasi Metropolis. These hospitals are named Hospital A, Hospital B, and Hospital C for anonymity. The first hospital (A) is public-funded while Hospitals B and C are private-funded hospitals.

### The population of the study and sampling technique

Women diagnosed with primary or secondary infertility and seeking treatment at the study sites were purposively recruited for the study. They accepted to be part of the study after the benefits and purpose of conducting the study had been explained to them. Informed consent was obtained from participants before being interviewed. The participants could express themselves fluently in the English language, Twi or Fanti dialects (two popular dialects in Southern Ghana which the lead author understands and speaks fluently). They were, at least, 21 years old. Data saturation was achieved with the nineteenth (19th) participant when no new themes emerged concerning the objectives of the study.

### Data collection

In line with the objectives of the study and in consultation with relevant literature, a semi-structured interview guide was developed to capture lived experiences of women with infertility. The interview guide was in two parts—sections A and B. Section A had questions on participants ‘personal data and clinical characteristics’ while Section B comprised questions that explored the emotional experiences, social experiences, coping strategies, and health-seeking behaviours of participants. The interview guide was reviewed by EAB and was pilot tested before its use in the study. The in-depth semi-structured interviews were conducted by the LAF in either English, Fanti, or Twi based on the preference of the participants.

The collection of participants' information commenced in June 2018 and was completed in August 2018. Each interview session lasted between 45 and 60 min. Before each session, participants were briefed on how the interview would be conducted, and they were informed that the interview would be audio-recorded. Participants who accepted to partake in the study were given a written consent form to complete. During the interview participants were asked to provide personal information such as age, educational background, occupation, marital status, and duration of infertility (years). This was followed by open-ended questions that were asked based on the interview guide. Probes such as “what do you mean by this statement” and “please can you explain further” were used to help provide clarity on the experiences of participants. Interview notes were taken during the fieldwork, and these covered observations such as participants’ body language, general livelihoods, and environmental factors. Data saturation was reached on interviewing the 19th participant.

All the interviews were audio-recorded and transcribed verbatim immediately after each session. Recordings were downloaded on a personal computer and also saved on a password-protected flash drive.

### Data analysis

The study employed Colaizzi's [22] thematic analysis which is a step-by-step process in the analysis of the data. This involved the search for and identification of common threads that extended throughout the interviews. Data were analyzed concurrently with data collection and were managed manually. Transcripts were read several times by team members to make meaning of participants' responses. During this period codes were identified, and similar codes were put together to form a cluster of themes to give a full description of participants’ lived experiences. The cluster of themes was then formulated into a statement of identification.

### Trustworthiness

A comprehensive description of the study design, methodology, setting, and a detailed background information presentation of participants was provided to allow for replication of the study and applicability of research findings to similar settings. Trustworthiness was achieved in this study through confirmability, credibility, authenticity, and dependability. Full understanding and accurate presentation of participants' narratives were achieved through member checking with four [4] participants. Field notes were written at each interview session. Also, the findings of the study were discussed among team members to enhance credibility.

### Ethical consideration

All methods were carried out in accordance with relevant guidelines and regulations. Ethics approval, with reference number CHRPE/AP/608/18, was obtained from the Committee on Human Research and Publication Ethics (CHRPE) of KNUST, Ghana. Approval was also obtained from the three hospitals (hospitals A, B, and C) where the interviews were conducted. Participants were informed that participation in the study was voluntary and they were at liberty to withdraw from the study at any time without any sanctions. Participants' anonymity and confidentiality were ensured through the use of pseudonyms. Consent forms were completed and participants signed.

## Results

The results are organized according to the demographic profile of informants (Table 1) and themes. Three main themes, with their subthemes, were obtained. Some illustrative quotations are also included in the text.

**Table 1:** Participants' Demographics

Participant	Age(years)	Level of education	Occupation	Duration of diagnosis (years)	Duration of marriage (years)
1	40	Primary	Trader	9	11
2	24	Secondary	Trader	2	3
3	26	Primary	Tailor	3	4
4	32	Tertiary	Teacher	9	10
5	29	Secondary	Trader	5	6
6	28	Tertiary	Store supervisor	2	4
7	31	Secondary	Trader	3	4
8	36	Tertiary	Teacher	6	7
9	45	Primary	Tailor	15	20
10	36	Secondary	Shop attendant	2	3
11	29	Secondary	Trader	4	6
12	38	Tertiary	Nurse	12	13
13	35	Tertiary	Police officer	2	5
14	35	Secondary	Trader	3	5
15	42	Tertiary	Teacher	6	7
16	31	Primary	Trader	1.5	4
17	34	Secondary	Trader	2	3
18	48	Secondary	Trader	13	15
19	26	Tertiary	Student	3	4

### Socio-Demographic profile of informants

Nineteen female participants were interviewed in this study. All participants were married and their ages ranged from 21 to 50 years. Four participants had received education up to primary level, eight to the junior high level while seven had tertiary education. Nine participants were traders, three teachers, one nurse, two seamstresses, three store supervisors, a shop attendant, and a police officer. Duration of diagnosis ranged from 2 to 15 years.

Three main themes were identified from the data gathered namely emotional experiences, social experiences and coping strategies (Table 2).

**Table 2:** Main themes and corresponding sub themes

Main themes	Sub-themes
Emotional experiences	General emotions Triggers of pain and sorrow Perceptions about life
Social experiences	Experiences in the matrimonial home Experiences with relatives Experiences with friends Societal pressure and influences
Coping strategies	Faith-based Herbal-based Biomedical/fertility clinic Child Adoption as an option

### Emotional experiences

Most participants described how they worry about their diagnosis of infertility most of the time. The feeling of not having a child after several years of marriage and the expectations from society and families on both sides caused them to worry a lot. They worried about when it would be their time to get pregnant and eventually deliver.

*I think about my infertility a lot because other people will be thinking about why I haven't given birth after a long time in marriage.* [2]

Some participants compared themselves with their friends and neighbours who married after them but had given birth. This made them feel that they had delayed so much, putting them on an emotional roller-costa.

*I got married before a neighbour but she gave birth almost immediately. Everyone is talking about me because I have kept long [in giving birth].* [3]

Most participants perceived infertility as a problem, with their happiness dependent on having a child.

*If I'll be happy in this world, I need a child. As a married woman, if you do not have a baby, you will not have any happiness in your life *[15]*.*

Participants, generally, worried persistently because of their diagnosis and this, they believed, had led to the development of conditions such as hypertension, loss of appetite, and even sexual problems. Sex, according to some participants, had lost its role in the marriage as it had not achieved its ultimate purpose of childbirth. This appeared to make the act unpleasant and painful for such women.

*I think a lot. When I got to the hospital, I was told my blood pressure was high. I'm desperate. It is more like many others who married after me have given birth. I'm not happy. Sometimes I'm moody *[18]

Some participants described events that gave them the impression that they had conceived at certain times, only to be disappointed later. Some of these events were a delayed menstrual cycle that lasted for months, weight gain and increased belly sizes which gave the impression that they were pregnant.

*What sometimes hurt is that my period does not come on the scheduled date, so I think I'm pregnant but then it [the period] comes later then I realize I am not pregnant* [9]

Participants described circumstances that triggered painful and/or sorrowful emotions about their diagnosis.

*When I am chatting with friends and they begin to talk about their children, how they talk about their children, sometimes, reminds me of my situation *[16]

Some participants also explained that they feel miserable when they see other women walking hand-in-hand with their children. These are sights that they perceived as lovely but were unable to have because of their diagnosis. Seeing such ‘lovely sights’ caused emotional despair for them as they were reminded of their challenges in having a child. Some participants ended up avoiding social gatherings where they would behold sights of other parents with their children while, in some cases, others questioned why they were having such challenges.

*It's nice to take your children out to have fun. Sometimes when I see things like these on TV, I ask myself “when shall I get my own?”. After we worship during Eid, I lock myself in the room alone, feeling I don’t have any happiness in my marriage and my life *[1]

Some participants described feeling awkward when questions about pregnancy were asked, making them view life as unfair.

*Life is not fair; I sometimes feel God shouldn't have created me *[2]

Some participants were convinced that they would have children in a matter of time and perceived their challenge as less severe, compared with the problems faced by other individuals/families in life.

*It’s a matter of time. Others have problems bigger than mine. For me I just thank God.*[7]

### Social experiences

Participants expressed mixed reactions about how infertility had affected their relationships with their husbands in their matrimonial homes. Some participants explained that nothing had changed in their matrimonial home.

*I live with my husband alone. Nothing has changed since the diagnosis; there has always been happiness in the home. My husband and I are still having a happy married life *[8]*.*

The second category of participants perceived differences in attitudes of their husbands, which they attributed to their infertility.

*He expected me to go to the hospital but I had resigned myself to the situation. So, he got angry because he thinks that it doesn't bother me *[4]*.*

Participants were mostly concerned that though their husbands were responsible and supportive, there was the possibility of them having children outside the marriage and this could lead to divorce. With the pressure that comes from parents and friends, they could be tempted by the idea of getting another spouse or ending the marriage.

*My husband says he loves me but the childlessness can cause a breakup *[16]*.*

Some participants explained that they were being pressurized by their own families, mostly their mothers, as a result of their infertility.

*My mother thought my husband was the cause but since he got another woman pregnant, she now thinks I am the cause. She advises that I find another man because our blood may not be compatible *[12]*.*

Participants explained that, sometimes, family members prevented their children from getting closer to them for reasons possibly related to their infertility. Other participants complained of the insolence of children of family members who lived with them and, whenever they were disciplined, they told their parents, causing further issues in the family.

*I brought my younger sister's child to live with me but whenever I discipline him, he goes to tell his mother and the mother is not happy about it *[9]*.*

Some participants also noted that they have had issues with their friends. They explained that their friends complained about their infertility and, in some cases, mocked them. Some were made to feel that they had no use for their wealth as they had no children that they had to cater to while others also felt blamed for not doing much to address their infertility issue.

*They [my friends] pass comments like 'what are you doing with money since you don't have a child'. They make it seem like you don't use the money for anything because you don’t take care of a child *[6].

Society became advisers, with a lot of people asking questions or seeking to provide possible solutions for participants. Some of these solutions proffered include the use of herbs, visits to hospitals, and visits to native doctors.

*Some people advise me to get help from native doctors and other sources. For instance, when I was living with my husband our landlord advised my husband to take me to see a certain native doctor but 1 refused. *[7].

Generally, participants noted that society expected married women to bear children within a year of marriage. People began to ask them questions when there was no sign of pregnancy or childbirth after the first year of marriage, and they were usually on the lookout for possible signs of pregnancy. Thus, any minor change in the physique or health of the woman was attributed to pregnancy, and participants felt pressured by these acts.

*Since everyone is just waiting for you to give birth or get pregnant, the moment you say you are ill or they see certain changes in you they think you are pregnant *[11]*.*

### Coping Strategies

The study found three main coping strategies that were employed by participants in dealing with infertility and its associated challenges – faith-based strategies, herbal-based strategies, and care from the biomedical setting (fertility clinic). Another coping strategy that was explored among the participants was child adoption but it did not receive favourable responses. All identified coping strategies were mainly driven by their hope that having a child of their own was a possibility despite all the prevailing circumstances.

It was their firm belief that God/Allah would work for them to have a child. This was usually utilized concurrently with seeking care at the fertility clinic and was mainly through fasting and prayers.

*I believe Allah will give me children when it's my time. It's just that, sometimes, some people make you think and worry about these situations. *[4]

Participants also mainly reported using herbal medicine before coming to the fertility centre when they realized that the approach was not helping in achieving their desired outcome.

*There was a woman who used to give me herbal medicine but it did not work so she recommended that I see the doctor *[19]***.***

*I was using herbal medicine. When it was not working, my sister recommended the hospital to me *[7]***.***

Participants mentioned some factors that caused them to seek care at the fertility centre. The first dominant factor was related to the perceived ineffectiveness or negative side effects of alternative medicines.

*The pain from menstruation made me come to the hospital. Had it not been for the pain, I would have continued with the herbal medicine *[8]

*I used herbal medicine for a long time. Before then I had been to the hospital and I was told my tubes were blocked. The doctor told me the only way I could conceive was either through a miracle or IVF* [4]

Another major factor was the attitude of the husbands of the women.

*The man I live with impregnated another woman and when I got to know, I realized my childlessness was a serious issue. He wasn't paying attention to me. He told me he will be with the one who is pregnant for him. And he warned me not to take any action that will bring untold issues. That is why I'm here *[15]

When child adoption was explored as a way of managing infertility, all the participants said they have heard of it, except one woman. However, most of them were not aware of the processes involved in child adoption and this was because participants were generally not prepared to consider child adoption as an alternative to having children. Some were firm in their responses to the extent that they mentioned that they would opt out if anyone talked or attempted to force them into it.

*“I have heard of it but I am not sure I will do it. I do not think I will ever become happy when I adopt a baby. I'll know it's not my child I'm living with *[1]*.*

There were several reasons why participants opposed the idea of child adoption. The first issue was related to possible problems that may arise, especially when the child finds out that he is not living with his/her biological parents. They were concerned that their behaviour towards the adopting parents may change as a result.

*I know of it [child adoption] but I don't like it. This is because someone will tell you he or she is not your child. My mum had one and when he grew up, he was told my mother was not his mother. And since then, he started misbehaving. It even got to a point he started stealing from the house *[12]*.*

*I have taken care of a lot of children that are not biologically mine, but when they grow, they leave to go find their biological parents *[9]*.*

According to some participants, adopted children were seen as outsiders by extended family members.

*I have heard of it, and I think it's good. However, people will say I've bought a child since they never saw me pregnant *[15]*.*

Again, participants were not sure of their husbands' behaviour towards the idea of adoption.

*It is not a bad idea but as I've told you I don't want to do anything that will offend my husband. Child adoption is something he doesn't like *[14]*.*

There was only one participant who was of the view that although she believed that childbirth is a gift from God, it would not be a bad idea to go for adoption as long as someone would call her mother.

*For me, I think adoption will be okay because if the person does not know his or her parents but will call me mother or will come and meet me when I am returning from work, I will be fine with it *[19]*.*

## Discussion

This study explored the lived experiences and coping strategies of women seeking treatment for infertility in three selected hospitals in the Kumasi Metropolis, Ghana. In research by Noorbala, and Ramezanzadeh [23] in Tehran, emotional disorders were found in 27.9% of couples with infertility. This study was consistent with their study which concluded that childlessness has a great influence on the emotional well-being of married couples with infertility, destabilizing affected women within their families and communities. Participants of this study reported emotional experiences such as fear, anxiety, and worry. These emotional experiences resulted in stress and loneliness. Infertility can be very painful for couples, but it has been pointed out that support from family, friends, and new treatment opportunities can alleviate all psychological discomforts.

According to Keskin, and Coksuer [24], infertility is a difficult problem for women regardless of the cause. It is important to note that most scholarly articles have combined the mental problems of childlessness and its medications. An article by Domar [13] pointed out that the majority of women with infertility felt depressed when in the company of pregnant women or couples with children. Similarly, participants in our study reported experiencing grief and hopelessness, especially when they saw other parents with their children.

Participants of this study reported that they worry about their situation a lot and, sometimes, keep to themselves. This finding relates to that of Nilsen and Waldenström [25] where women acknowledged being worried about their infertility problem. In a similar study by Farzadi, and Mohammadi-Fosseini [26], the majority of the participants reported succumbing to serious sadness and dissatisfaction due to their inability to become pregnant. The participants said they cry always as a result of their situation and felt that it helped them to deal with their unfulfilled desires [27].

After realizing that they are not able to bring forth a child into the world, many couples go through emotional stress related to infertility [28]. Such stressors include dynamism in the network of their families and society, interpersonal relationship quality, and changes in enduring their predicament. These were not reported by the participants of this study. They, however, reported avoiding social activities, some relatives and friends.

In Pakistan for instance, women with infertility are perceived as carriers of bad luck and are prevented from attending crucial societal events such as marriage ceremonies and outdooring celebrations. They are treated with disdain and are not allowed to interact with others’ children [29]. Our study revealed that the Ghanaian society does not perceive people with infertility as carrying bad omen nor are they prevented from attending social events. However, they are looked down upon in some instances and are seen as individuals who have fewer responsibilities and as such do not have so much financial support. Nevertheless, the majority of the participants said they, sometimes, keep themselves busy at work and involved in activities that make them forget about the problem of infertility.

Another aspect that bothered some women in this study was the strained relationships with their husbands, with some living with a constant fear that their husbands/partners may go in for other women if the infertility challenge persists. It seems generally acceptable for males to have many other options for coping, including marrying or giving birth with another woman outside the marriage. This is however not so in the case of women [30], and this adds to their worries.

Expectations from society also play a part in the desire to conceive, and where childbearing may be a socio-cultural norm, inadvertently couples without children feel more separated by their circumstances [31]. The findings of the present study showed that childlessness was a vital source of social problems for most of the participants. This issue could elevate important social problems in the lives of women with infertility.

A comprehensive view of the literature shows that women in some countries with infertility are sometimes seen as misfortune and this leads to being despised by other people [32]. As indicated in the results, childlessness produced some negative experiences for women in their matrimonial homes. For most participants, feuds were evident in their matrimonial homes but did not result in any significant violence or conflict with their partners. This finding contradicts studies in other parts of Africa and some Asian countries including Egypt, Pakistan, Kuwait, Turkey, and Iran where some individuals with infertility, especially women, suffered domestic physical violence and verbal violence from people around them [32, 33]. Most of the participants in the current study did not have any negative experience largely from their husbands in their matrimonial homes. Although the behaviour of husbands was seen to be that of despair and frustration in this study, this did not generally affect their level of care or responsibility shown towards their spouses. The majority of the husbands were described as caring and supportive. Thus, many of the study participants were not pressurised by divorce or remarriage by their husbands in particular as reported by studies elsewhere [34].

Participants were, however, concerned that although their husbands may be responsible, there was also the possibility of them having a child outside the marriage because they may also be frustrated with the situation. Furthermore, in many developing countries such as Ghana, the man is generally considered to have a right to marry another wife if there are challenges in having a child. From the study, it was a big problem for many participants to discuss their issues with neighbours. Considering Ghana’s strong tradition of large families [35], it is reasonable to expect that couples who are encountering fertility issues might feel being avoided by their family members due to the prolonged delay to procreate as the society is seen to be more expectant of a baby being born after marriage. Moreover, some women experience some levels of hostility from their in-laws as a result of their condition. Bruner [36] explains that most families become hostile toward the woman because they perceive that infertility is not the fault of the man but the woman.

The findings of this study suggest that there is the potential for stigma and accusation from the community as well as the relatives of women with infertility. These kinds of attitudes tend to trigger painful emotions in them. This finding corroborates a study by Ramezanzadeh, and Aghssa [37] among women diagnosed with infertility who were undergoing treatment. It was said that the women were anxious due to some of the utterances made by their peers such as comparing them with their mates who got married in the same year.

There's a noteworthy distinction in infertility between developing and developed nations [38]. Among developing nations, the capacity to procreate depends on the couple's interest, particularly among ladies [32]. In developed societies, be that as it may, deliberate childlessness is seen as a more reasonable and true-blue alternative, and ladies without children are regularly assumed to be intentionally childless [38]. Therefore, the experiences of women with infertility with friends, families and the society that equates femininity with motherhood differ across cultures. Creating unnecessary comments can destroy and change the development of femininity [39]. In societies where there's no concept of intentional childlessness, it is inconceivable to cover up infertility. Problems of infertility, subsequently, may be more prominent in developing nations such as Ghana [40]. This finding ties in with that of Latifnejad Roudsari, Allan [41]. In their study, women with infertility tried to live peacefully by adopting coping strategies. D’Souza Vinitha, and Shobha [41] carried out research in the United States and identified that infertile women used different coping strategies that help them to cope with the problem of childlessness.

All coping strategies mentioned in this study were used by participants except for child adoption. Most of the women in this study were not prepared to consider child adoption as an alternative to having children and the reasons were in relation to the culture and societal expectations. Indeed, studies have shown that child adoption is an unpopular decision in India Bharadwaj [42]. This suggests that cultural and societal expectations influence coping mechanisms women with infertility may adopt in different settings. Regarding social support, Boivin et al. [2] concluded that the most common support resources were discussions with spouses, family members, and friends and the use of material about the psychological aspects of childlessness from health sectors, the press, or television programs Thus, it is stated that social support performs an active role in the management of infertility [43]. Yet, in a study in South Africa, several of the women did not want to speak to others about their involuntary childlessness because they want to hide their identities [40].

Harris and Molock [34] distinguished that whereas numerous women found that talking to others around their childlessness demonstrated accommodating, others ladies did not talk to anybody other than their companions because they were more concerned about their reputation after disclosure [44]. Johnson and Fledderjohann [45] also found that most women were concerned that telling others about their issues of infertility would pave way for them to be mocked. Although some participants of this study drew on similar social support systems, these were also considered a source of stress for others. In any case, a few of the participants were concerned that the great relationship they were having with their spouses might be altered if the infertility issue persists.

Some studies concluded that women with infertility have communicated comparative assumptions in feeling backed by their spouses [20, 49]. Boivin et al. [2] determined that discussing with one’s companion also serves as a source of support. Therefore, women with infertility adapted to their issues by sharing sentiments in connection with childlessness with their spouses. Women with infertility must get help from their husbands because lack of support is a key source of anxiety and depression. Komaromy [46] concluded that individuals sought medical help and became knowledgeable about infertility in trying to cope with infertility. More than half of the participants in this study did not actively read or learn about infertility through other means other than solely relying on healthcare professionals for information. This may be because some of the participants were not highly educated. Most of the participants in this study viewed their religious faith as means of adapting to their childlessness issues. This is in line with another study that reported that numerous women depended on their solid devout convictions and changed devout hones in tending to their fertility issues [41]. All participants communicated devout conviction as a vital source of backing.

Participants were also assessed on factors affecting health-seeking and how they got to know about the health facility they were seeking care from. Although the use of herbs for the management of infertility was welcomed among rural folks in the southern part of Ghana [47], and the availability of local herbal drugs makes it very difficult for women diagnosed with infertility to seek biomedical care [48], some participants talked about the ineffectiveness or negative side effects of these approaches. Most Ghanaians tend to follow recommendations of herbal centres from friends or relatives than any other source, making peer-to-peer marketing very powerful [49]. It was, therefore, not surprising that many women utilized herbal-based approaches to resolving their problems with infertility before reporting to their respective fertility centres.

The outcome of the study indicates that mindfulness is likely to assist women with infertility in getting viable restorative care, complying with treatment, and bargaining with their circumstances. Shockingly, some of these women, out of numbness and need of direction, resort to the utilization of strange measures such as the utilization of concoctions [38]. The importance of health education and counseling is recognized, and both need to be integrated into infertility management. This will help eradicate some wrong practices such as the use of concoctions that can be harmful to the reproductive system of women.

## Limitations of the study

The study had some limitations. First, the study participants comprised persons who had decided to seek assisted reproductive treatment. Experiences and coping strategies of persons with infertility not seeking biomedical care at the fertility clinics could, therefore, not be explored in this study. The second limitation of this study was that the participants were interviewed at the various centres and that could have influenced what they said. But in all of these, the participants were reassured not to feel intimidated by whatever reason, because the exercise was purely for research purposes.

Moreover, the non-involvement of husbands (spousal disengagement) in this study was a major limitation because their perspective would have been important to allow for a conclusion that would represent both husband and wife as an entity.

## Conclusions

Infertility is a paramount issue for couples living with it in the Ashanti Region of Ghana. The pattern of responses to questions asked and the kind of emotions that were expressed reflected their encounters.

The main conclusion drawn from this study is that women diagnosed with infertility are usually in great psychological distress and experience social withdrawal. Future research could evaluate not only interventions with these indicated components but also determine with whom such interventions would be most effective to understand the complexity of factors that have to do with infertility among couples in the Ashanti Region and other parts of the country (Additional file 1).

## Supplementary Information

**Additional file 1**. Interview Guide.

## Data Availability

Data analyzed during this study may be available from the corresponding author upon reasonable request.

## Abbreviations

ART: Assisted reproductive technology
IVF: In -vitro fertilisation
GIFT: Gamete intra-fallopian transfer
ZIFT: Zygote intra-fallopian transfer
