# Supplementary material for: Lived experiences and coping strategies of persons seeking infertility treatment in the Kumasi metropolis: a descriptive phenomenological study
Source: BMC Womens Health. 2023 Feb 20;23:74. doi: 10.1186/s12905-023-02194-6 (PMC9940398; doi:10.1186/s12905-023-02194-6)
Supplement: Supplementary file 1 — Additional file 1. Interview Guide. [file 12905_2023_2194_MOESM1_ESM.pdf]

APPENDIX

INTERVIEW GUIDE

Section A

Personal Data:

Age	[ ]
Sex	[ ]
Occupation	[ ]
Religion	[ ]
Tribe	[ ]
Education	[ ]

Section B

Psychological experiences

1. Please tell me how you felt when you were diagnosed with infertility?

2. As an individual diagnosed with infertility, how have you been feeling deep within?

3. As a married person with infertility, what issues/problems does this situation bring?

4. What reminds you of this situation?

5. What are normally your feelings or reactions as you remember?

6. How do you see life in this situation?

Social Experiences

1. Can you please share with me how marital life is with respect to your condition?
2. Considering the Ghanaian culture which attaches so much importance to having your own biological children, what are some of your experiences in relation to your:

- Relatives
- Husband
- His Relatives
- Friends

3. From your experiences, how does society look at you?

4. From your understanding of the situation, how will you compare your position in the Society before and after the diagnosis?

5. Can you kindly describe how you relate with people before and after the diagnosis?

Coping strategies

1. Considering all that you have told me, have you been using some measures to Adjust?

2. If no why?

3. If yes, can you please share with me those measures?

4. What do you think of child adoption as a treatment option?

5. Do you have any idea of the adoption process?

Health-seeking behaviour /biomedical /fertility clinic

1. Can you share with me the general situation regarding your seeking for help?

2. What are the factors that entreated you to come to the hospital for help?

3. Have you ever tried using other ways of treatment before coming to the hospital?

4. Were you advised by someone to come to the hospital or you made the decision by yourself?
